# Non-occlusive mesenteric ischemia localized in the transverse colon: a case report

**DOI:** 10.1186/s40792-017-0299-x

**Published:** 2017-02-10

**Authors:** Koji Murono, Soichiro Ishihara, Kazushige Kawai, Manabu Kaneko, Kazuhito Sasaki, Koji Yasuda, Kensuke Otani, Takeshi Nishikawa, Toshiaki Tanaka, Tomomichi Kiyomatsu, Keisuke Hata, Hiroaki Nozawa, Akimasa Hayashi, Tetsuo Ushiku, Masashi Fukayama, Toshiaki Watanabe

**Affiliations:** 1 Department of Surgical Oncology, Faculty of Medicine, The University of Tokyo, Tokyo, Japan; 2 Department of Pathology, Graduate School of Medicine, The University of Tokyo, Tokyo, Japan

**Keywords:** Non-occlusive mesenteric ischemia, Colon, Report of a case, Computed tomography angiography

## Abstract

**Background:**

Non-occlusive mesenteric ischemia (NOMI) is ischemia of the mesentery that is caused by hypoperfusion or vasospasm without any thrombosis. NOMI is difficult to diagnose by physical examination alone. Although angiographic examination of the superior mesenteric artery (SMA) is the usual diagnostic method used, it is an invasive examination. Usually, a long range of the bowel becomes discontinuously necrotic in NOMI. Here, we report a rare case of NOMI localized in the transverse colon that was diagnosed by computed tomography (CT) angiography which is a minimally invasive examination.

**Case presentation:**

A 72-year-old woman was referred to our hospital for further treatment of abdominal pain that developed 1 day before presentation. Contrast-enhanced abdominal CT scan revealed attenuated enhancement of the transverse colon. CT angiography showed SMA irregularities due to vasospasm. The middle colic artery could not be detected by CT angiography. No occlusion due to thrombus or embolism in the SMA and superior mesenteric vein was observed. Based on the findings, NOMI was suspected, and emergency laparotomy was performed, which revealed a segmentally necrotic transverse colon. The necrotic bowel was resected, and stomas were created.

**Conclusion:**

The presence of hypotension in the patient necessitated the use of CT angiography, which proved very useful in the early diagnosis of the present case. Thus, if intestinal ischemia is suspected, even in case of a short segment, CT angiography should be performed.

## Background

Non-occlusive mesenteric ischemia (NOMI) is ischemia of the mesentery without thrombus or embolism in the superior mesenteric artery (SMA) or vein (SMV). It is caused by hypoperfusion or vasospasm and was first reported by Ende et al. in 1958 [1]. Hemodialysis, diabetes mellitus, advanced age, hypertension, and low cardiac output are the risk factors of NOMI. Diagnosing NOMI is difficult because the abdominal pain it brings is often mild and the symptoms are non-specific [2]. Thus, the mortality rate remains very high (45–59%) [3–5].

In patients with NOMI, a long range of the bowel usually becomes necrotic, and typically, skip lesion is observed, which necessitates the resection of a large amount of the necrotic bowel. Here, we report a rare case of NOMI localized in the transverse colon that was diagnosed by CT angiography.

## Case presentation

A 72-year-old woman, who had hypertension and emphysema treated by her personal physician, was referred to our hospital for further treatment of abdominal pain, which developed 1 day before presentation. Oral intake was decreased because of the abdominal pain. The pain was mild, and no peritoneal irritation sign was detected. The vital signs were normal, except for the blood pressure, which was low (88/59 mmHg). Dehydration was suggested owing to the elevated level of blood urea nitrogen and serum creatine. Although her lactate dehydrogenase and creatine kinase levels showed no abnormality, white blood cell count and aspartate aminotransferase level were elevated (11,600/μL and 88 U/L, respectively). Moreover, metabolic acidosis was observed (pH, 7.211; HCO_3_, 12.7 mmol/L; base excess, −13.8 mmol/L).

The patient underwent 3D-CT angiography using a 64-detector CT scanner. Iopamidol (370 mg I/mL, Iopamiron 370; Bayer, Osaka, Japan) was used as the contrast agent. The patient was injected with 0.7 g/kg iodine for 30 s, and the scan timing was 90 s after the injection. The tube potential was 120 kVp, and the tube current was adjusted by automatic exposure control with a noise index of 10 and a slice thickness of 0.5 mm. Image processing analysis was performed using a 3D volume rendering technique with the ZIOstation system (Ziosoft, Tokyo, Japan).

**Fig. 1:**
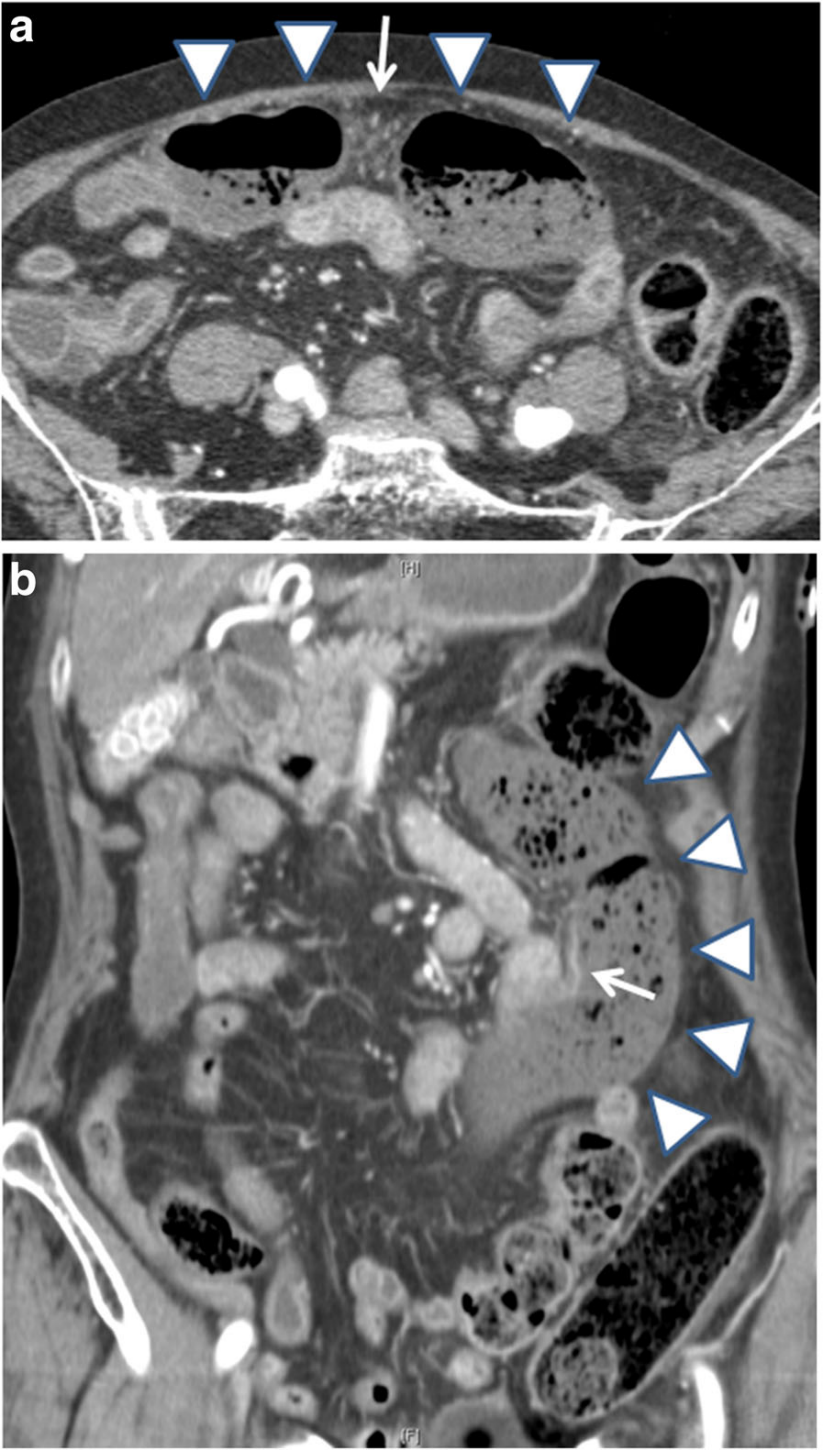
Contrast-enhanced computed tomography scan. **a** The dilated and thinned transverse colon was detected in the axial view. The enhancement of peripheral artery was not attenuated (*arrow*). **b** Remaining partial enhancement of the transverse colon (*arrow*). The enhancement of the other part of the transverse colon was attenuated

**Fig. 2:**
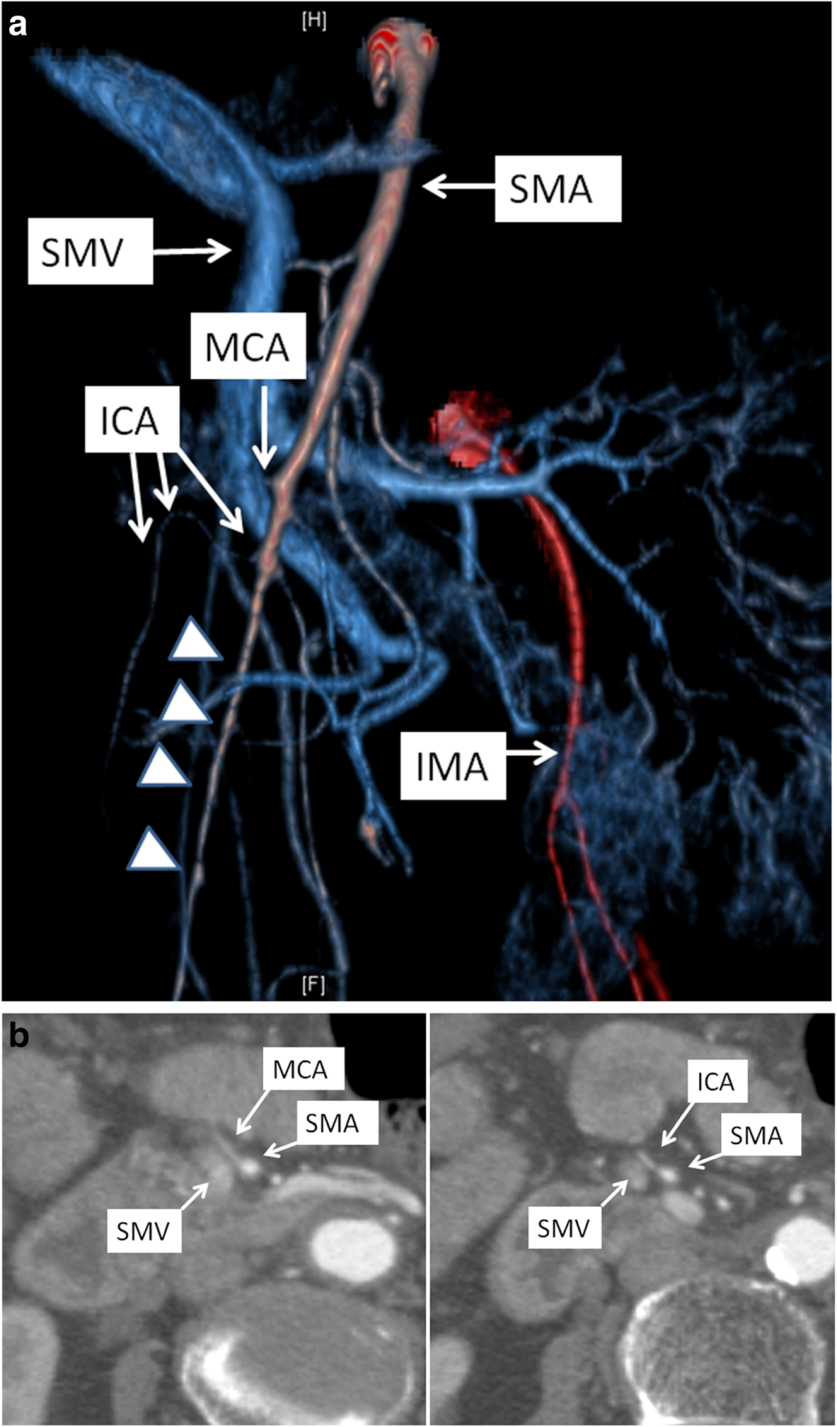
**a** Front view of the CT angiography. The irregularities of the SMA due to vasospasm were observed. The MCA could not be detected by CT angiography, and the narrowing of the ICA was observed. The RCA could not be detected. Although the IMA was patent, the left colic artery was also undetectable. There was no abnormality in the SMV. **b** In 2D axial view, compared to the SMV, narrowing of the SMA, MCA, and ICA was observed. No thrombosis was detected in the SMA and SMV. *ICA* ileocolic artery, *MCA* middle colic artery, *IMA* inferior mesenteric artery, *SMA* superior mesenteric artery, *SMV* superior mesenteric vein

Contrast-enhanced abdominal CT scan revealed a dilated and thinned transverse colon. The enhancement of the transverse colon was mainly attenuated, except for a remaining partial enhancement (Fig. 1). CT angiography showed irregularities of the SMA due to vasospasm (Fig. 2). Narrowing of the middle colic artery (MCA) and ileocolic artery (ICA) was observed by 2D axial view, and the MCA could not be detected by CT angiography (Fig. 2). The right colic artery (RCA) was also undetectable, which suggests the lack or occlusion of the RCA. Similarly, the left colic artery (LCA) was not detected. No occlusion of the SMA and SMV due to thrombus or embolism was observed, and the peripheral arteries of the transverse colon were also enhanced.

**Fig. 3:**
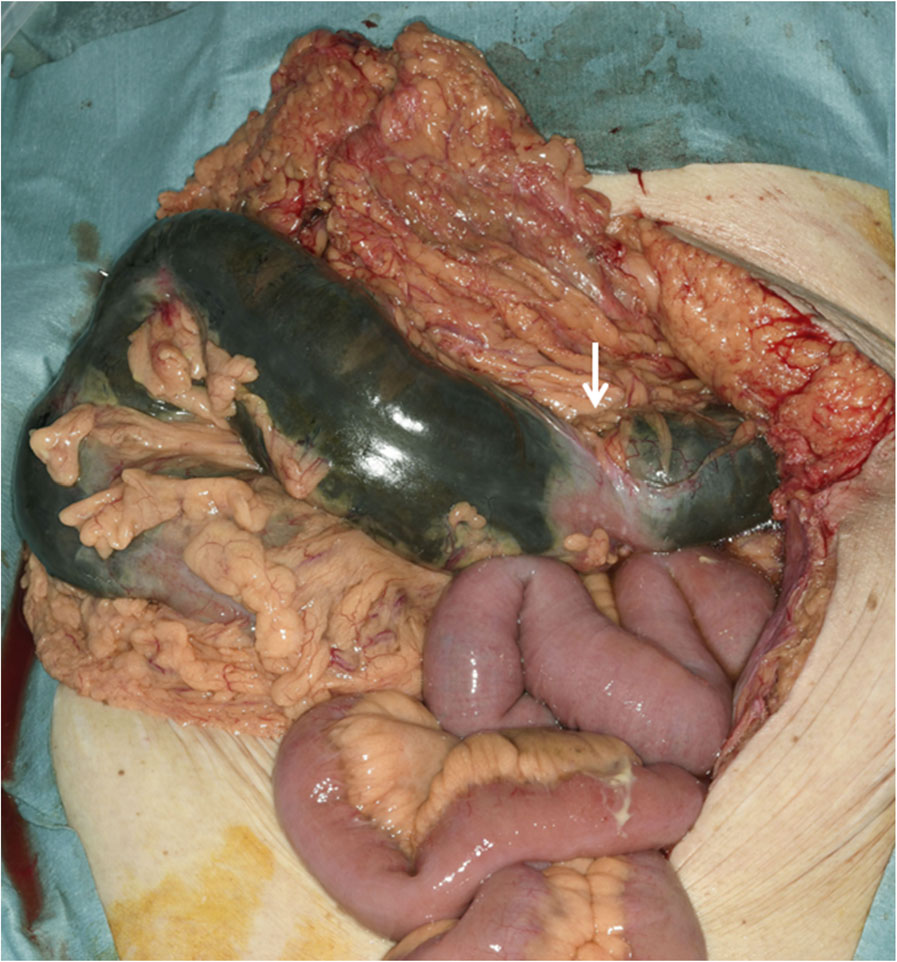
Intraoperative colon findings. The transverse colon became discontinuously necrotic (the normal bowel was indicated in *arrow*), and the pulsation of peripheral arteries of transverse colon was not attenuated. The necrotic transverse colon was resected

Based on these findings, necrosis of the transverse colon due to NOMI was suspected, and emergency laparotomy was performed. At laparotomy, the transverse colon was segmentally necrotic, as diagnosed preoperatively; however, the pulsation of the peripheral arteries of the transverse colon was not attenuated. Intraoperative findings also suggest NOMI, and the necrotic bowel was resected (Fig. 3). Bowel anastomosis was not performed, and stomas were created.

**Fig. 4:**
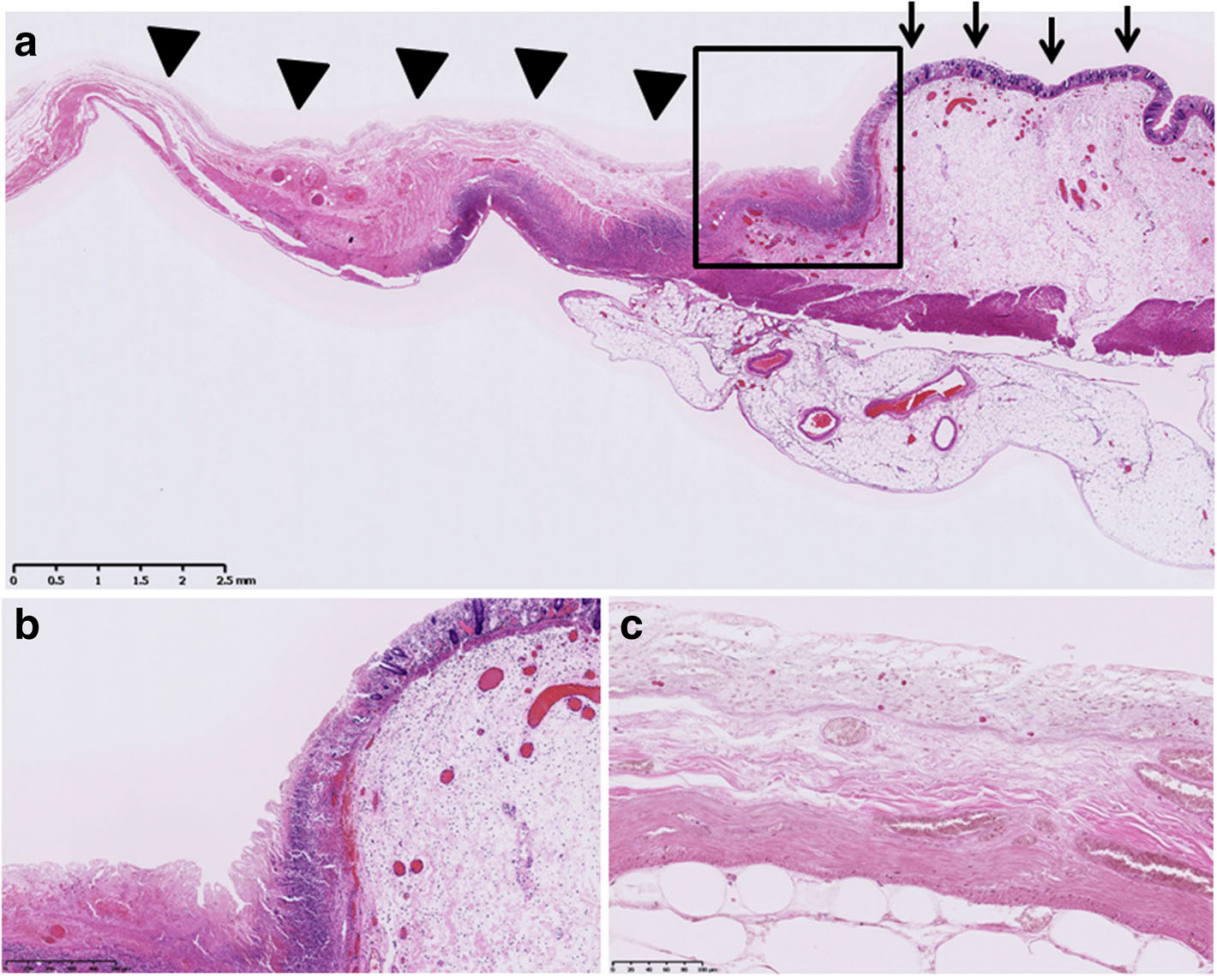
**a** Various depths of necrosis of the intestinal wall. Ischemic change with mucosal atrophy and submucosal edema is indicated by *arrows*. Regional transmural necrosis with lack of the mucosa and thinning of the intestinal wall is indicated by *triangles*. **b** Close-up image of the *square* in **a**. Ghost-like disappearance of the mucosal glands was identified in the borderline region. **c** Close-up image of the necrosis of all layers of the intestinal wall. Massive enucleation of smooth muscle cells was observed. No thrombus was identified (nor confirmed in **a**)

Histopathological findings showed massive ischemic changes with various depths of necrosis, ranging from mucosal ischemia to transmural necrosis, which reflect discontinuous necrosis of the transverse colon (Fig. 4). Arterial or venous thrombus was not identified in the resected specimens, which also supported NOMI. The patient’s postoperative course was uncomplicated, and she was discharged from the hospital on postoperative day 33.

## Discussion

Angiography of the SMA is necessary for the diagnosis of NOMI. Angiographic findings suggestive of mesenteric vasospasm are (1) narrowing at the origins of the major branches of SMA, (2) irregularities in the intestinal branches with segmental narrowing, (3) spasm of the intestinal arcades, and (4) impaired filling of intramural vessels [6]. After mesenteric vasospasm is confirmed by angiographic examination, prostaglandin or papaverine hydrochloride injection generally improves the vasoconstriction [7]. However, angiography is an invasive and time-consuming examination that cannot be performed in cases with serious conditions such as severe hypotension.

CT is often used to diagnose NOMI by detecting the signs of intestinal ischemia such as attenuated enhancement of the intestinal wall, dilated intestine, and intramural gas [7–10]. Vasospasm of the SMA, as indicated by the SMA diameter and through analysis on multi-planar reconstructed (MPR) images, was also reported as a diagnostic finding of NOMI [8, 9], which was detected in this case. However, the usefulness of CT angiography has not been evaluated thus far. In the present study, angiographic examination was difficult to perform because of hypotension, and the ability of CT angiography to detect SMA irregularity and narrowing of the origin of the MCA and ICA was very useful in the early diagnosis.

Although there are only a few reports on the pathological features of NOMI, Sarda et al. reported congestion and flattening of the plicae with inflammatory exudates in the substantia propriae in less advanced cases as well as necrosis and ulceration of the mucosa, submucosa, and muscularis. Typical macroscopic findings show well-defined multiple circumferential ulcers of variable depths. In the present case, congestion and flattening of the mucosa and edema of the submucosal layer were observed [11]. Although no multiple ulcers were detected, there was necrosis of variable depths with clear margin and regional transmural necrosis with peripheral ghost-like disappearance of the mucosal glands was identified. These ischemic changes and the absence of thrombus in resected specimens were consistent with NOMI.

Ischemic colitis is also known to sometimes cause bowel necrosis despite the absence of thrombosis or embolism in the mesenteric arteries. In many studies, NOMI was not distinguished from ischemic colitis. Wittenberg et al. reported that NOMI typically occurs in the area of the bowel perfused by the SMA, whereas ischemic colitis usually presents in the area perfused by the inferior mesenteric artery [12]. Usually, a long range of the bowel becomes discontinuously necrotic in a patient with NOMI. Intraoperative endoscopy is useful for the evaluation of extent of mucosal necrosis. However, because of the risk of contamination of operation field, extended mucosal necrosis was evaluated by the inspection of the resected specimen in this case. No extended mucosal necrosis was observed in the present case.

Only a few cases of NOMI localized in the colon were reported [7, 12], and the ascending colon was the most frequently reported site. There was no report of a case localized in the transverse colon. Although narrowing of the ICA was observed in the present case, the narrowing of the MCA was more severe than that of the ICA, and the blood flow of the MCA was not detected by CT angiography (Fig. 2). Moreover, in the present case, the LCA could not be detected by CT angiography, which suggested the absence or occlusion of the LCA (Fig. 2), although we could not confirm that during the emergent operation. In our previous study, the absence of the LCA was observed in only 5.1% of the cases [13]. The absence of the LCA, which could be a collateral artery of the transverse colon, may contribute to this rare case of ischemia localized in the transverse colon. The descending colon was not ischemic, which implied that the sigmoid artery assumed the role as a collateral artery. Being less invasive, CT angiography was superior to traditional angiographic examination in acquiring information on multiple arteries.

## Conclusions

NOMI is difficult to diagnose by physical examination alone, such as in the present case. If intestinal ischemia is suspected, even in case of a short segment, CT angiography should be performed as a minimally invasive examination.

## Abbreviations

CT: Computed tomography
ICA: Ileocolic artery
LCA: Left colic artery
MCA: Middle colic artery
NOMI: Non-occlusive mesenteric ischemia
SMA: Superior mesenteric artery
SMV: Superior mesenteric vein
